# Gas-Source CVD Growth of Atomic Layered WS_2_ from WF_6_ and H_2_S Precursors with High Grain Size Uniformity

**DOI:** 10.1038/s41598-019-54049-6

**Published:** 2019-11-27

**Authors:** Mitsuhiro Okada, Naoya Okada, Wen-Hsin Chang, Takahiko Endo, Atsushi Ando, Tetsuo Shimizu, Toshitaka Kubo, Yasumitsu Miyata, Toshifumi Irisawa

**Affiliations:** 1 Nanomaterials Research Institute, National Institute of Advanced Industrial Science and Technology (AIST), 1-1-1, Higashi, Tsukuba, Ibaraki 305-8565 Japan; 2 Nanoelectronics Research Institute, National Institute of Advanced Industrial Science and Technology (AIST), 1-1-1, Umezono, Tsukuba, Ibaraki 305-8568 Japan; 3 Department of Physics, Tokyo Metropolitan University, 1-1, Minami-Osawa, Hachioji, Tokyo 192-0397 Japan

**Keywords:** Nanoscale materials, Nanoscale materials, Chemical synthesis

## Abstract

Two-dimensional (2D) transition-metal dichalcogenides have attracted a considerable amount of attention because of their potential for post-silicon device applications, as well as for exploring fundamental physics in an ideal 2D system. We tested the chemical vapour deposition (CVD) of WS_2_ using the gaseous precursors WF_6_ and H_2_S, augmented by the Na-assistance method. When Na was present during growth, the process created triangle-shaped WS_2_ crystals that were 10 μm in size and exhibited semiconducting characteristics. By contrast, the Na-free growth of WS_2_ resulted in a continuous film with metallic behaviour. These results clearly demonstrate that alkali-metal assistance is valid even in applications of gas-source CVD without oxygen-containing species, where intermediates comprising Na, W, and S can play an important role. We observed that the WS_2_ crystals grown by gas-source CVD exhibited a narrow size distribution when compared with crystals grown by conventional solid-source CVD, indicating that the crystal nucleation occurred almost simultaneously across the substrate, and that uniform lateral growth was dominant afterwards. This phenomenon was attributed to the suppression of inhomogeneous nucleation through the fast and uniform diffusion of the gas-phase precursors, supported by the Na-assisted suppression of the fast reactions between WF_6_ and H_2_S.

## Introduction

Two-dimensional (2D) materials, such as graphene, hexagonal boron nitride (hBN) and transition-metal dichalcogenides (TMDs), have gained considerable attention in recent few years, due to their electronic and optoelectronic properties^1–3^. Since monolayer group-VI TMDs, such as MoS_2_ and WS_2_, are flexible direct-gap semiconductors^3,4^, they are considered promising candidates for use in next-generation semiconductor devices, including extremely thin-body transistors, flexible electronic devices, photodetectors and light-emitting devices^5–9^. Monolayer TMDs can be prepared using a range of techniques, such as mechanical or chemical exfoliation of bulk single crystals, chemical vapour deposition (CVD) and molecular beam epitaxy^10–14^. The CVD method, in particular, is extensively used to fabricate large-scale monolayer TMDs with better reproducibility than that afforded by other methods. Typical CVD growth methods for TMDs use a metal oxide, such as MoO_3_ or WO_3_, and elemental sulphur as precursors, by heating the solid precursors along with a substrate under an inert gas atmosphere at a temperature above 500 °C. TMD monolayers with grain sizes larger than 10 μm have been prepared using this approach^15–17^.

One topic of recent importance for the CVD growth of TMDs is the ‘alkali-metal assistance’ method. When alkali-metal compounds, such as NaCl and KBr, are added into a solid-source CVD system, TMD grains are able to grow much larger than they otherwise would^18–24^. Because this effect is valid not only for group-VI TMDs, but also for other TMDs, such as NbS_2_ and TiS_2_^25^, gaining insight into the underlying mechanisms of this phenomenon is important for expanding the range of future applications of TMDs. Although the effect of alkali metals has not been completely clarified in terms of the chemical processes involved, several models have been proposed to explain the phenomenon. For example, the volatile metal oxychloride species and/or eutectic intermediate compounds generated during the process, such as Na_2_MoO_4_, could be affecting the chemical reaction route and enhancing the diffusion of the metal precursors^23–28^. Another hypothesis is that a catalytic effect at the edges of the forming TMDs may be promoting 2D crystal growth^22,29^.

One persistent problem encountered during the conventional CVD method is the low vapour pressure of the solid-source precursors, such as sulphur and metal oxides. Metal oxides (e.g. WO_3_, which is commonly used for growing WS_2_) have low vapour pressures at typical growth temperatures because of their high melting points (e.g. 1473 °C for WO_3_). This high melting point makes it difficult to control the precursor supply and achieve uniform, large-scale growth of TMDs^30^, meaning that solid-source CVD is not suitable for industrial TMD production. To address this issue, researchers have used the metal–organic (MO) CVD method, which uses MO compounds with a vapour pressure higher than those of conventional metal oxides and elemental sulphur. This approach has led to the successful growth of TMD grains^30–33^. Some studies have found that an alkali-metal assistance method is useful for MOCVD growth, where the addition of alkali-metal compounds makes grain sizes larger than that of alkali-metal-free growth^30,33^.

Another solution for improving a precursor’s vapour pressure is the use of gaseous materials as precursors. For example, researchers have reported success with CVD and atomic layer deposition (ALD) growth of WS_2_ using hydrogen sulphide (H_2_S) and tungsten hexafluoride (WF_6_) as precursors^34–39^. These precursors are in the vapour phase at room temperature and ambient pressures, so their supply rate can be precisely controlled via a mass-flow controller (MFC). Furthermore, they have simpler elemental compositions than MO precursors, and they do not contain carbon. As a result, the chemical reactions required for gas-source CVD growth should be much simpler than those encountered during MOCVD, and the possible carbon contamination issues of MOCVD growth can be avoided^32^. The use of H_2_S and WF_6_ provide a number of additional advantages over solid precursors: i) gas-phase supply makes large-scale growth easy (e.g. WS_2_ CVD and ALD growth from these precursors onto 300-mm Si wafers with a dielectric coating^35–38^); ii) they are already widely used in semiconductor manufacturing (e.g. WF_6_ is used for low-temperature CVD growth of W films on Si wafers to form W-plugs^40–42^) and iii) their high reactivity allows low-temperature WS_2_ growth (250–450 °C), which enhances their applicability^34–39^. With all of these advantages, gas-source CVD growth is a promising method for TMD mass production. However, the typical WS_2_ grain size obtained from these precursors is up to 200 nm^38^ which is much smaller than those derived from solid-source approaches, and studies aimed at increasing the grain size achievable with these highly reactive, oxygen-free precursors (e.g. through alkali-metal assistance) has not been reported.

Here, we report the CVD growth of WS_2_ atomic layers from H_2_S and WF_6_ precursors with micrometre-scale, highly-uniform grain size. We found that alkali-metal assistance method is still valid for a WS_2_ CVD growth form H_2_S and WF_6_ precursors: by introducing a NaCl, which is the most common alkali-metal compound into a CVD chamber, we obtained monolayer to bilayer WS_2_ with grain size of as large as ~10 μm on an oxidized silicon (SiO_2_/Si) substrate. This is tens of times larger than that obtained from NaCl-free growth and previous reports, and comparable to that of WS_2_ obtained from solid-source growth. The WS_2_ prepared with NaCl assistance exhibits n-type semiconductor behaviour, with an on/off ratio of ~10^4^ in field-effect transistor (FET) operation while a NaCl-free grown WS_2_ does not show any semiconducting behaviour. These results indicate that alkali-metal assistance is effective even for the case of using very-high reactive, gaseous precursors without oxygen like our sources. And simple elemental composition of the precursors allows us to simply discuss the effect of Na, that is, Na can suppress elemental W and WS_2_ cluster formation as nuclei during the growth. Furthermore, we found that NaCl-assisted WS_2_ showed very narrow grain size distribution with a standard deviation as small as 0.6 μm, which is much smaller than that of TMDs prepared by using solid precursors onto SiO_2_/Si substrate. This narrow size distribution would originate from high diffusive nature of gas-phase precursors allowed by alkali-metal assistance, where the WS_2_ nuclei form simultaneously and that further nucleation during 2D lateral growth afterward is strongly suppressed even on SiO_2_/Si substrates with abundant diffusion barrier such as dangling bonds and surface roughness, thereby demonstrating the feasibility of precisely controlling grain size over a wide area.

## Results

### Performance of NaCl-assisted gas-source CVD

**Figure 1:**
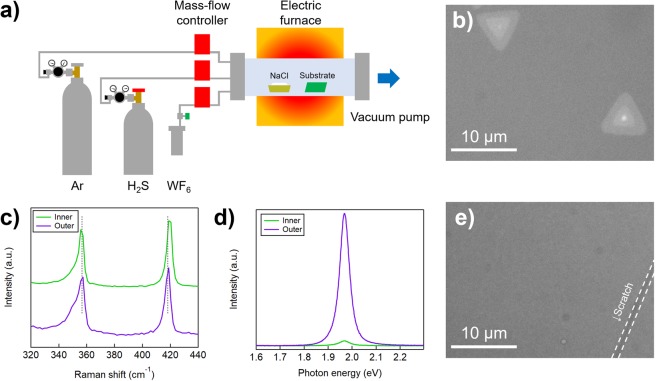
(**a**) A schematic of our CVD setup for the synthesis of WS_2_. (**b**) An optical microscope image of NaCl-assisted WS_2_. (**c**) Raman and (**d**) PL spectra of WS_2_ grown with NaCl. The dotted lines in (**c**) correspond to the peak positions of the E′ (356.7 cm^−1^) and A′_1_ (418.2 cm^−1^) modes in the monolayer region of WS_2_. (**e**) A typical optical image of WS_2_ grown in the absence of NaCl. The white dotted lines show the boundary between the scratch and the WS_2_ film. In (**b–e**), the green component was extracted from the original images and the contrast was enhanced for easy visualisation of WS_2_.

Figure 1a shows a schematic of our CVD setup. We used pure H_2_S and WF_6_ gases as precursors and Ar as a dilution gas. These gases were supplied directly from their respective cylinders to the CVD chamber via an MFC. The concentrations of these precursors were precisely controlled via the MFC, which was more precise than that of the solid precursors. NaCl powder was placed on the upstream side of the substrates in the chamber.

Figure 1b shows a typical optical image of the WS_2_ grains grown with alkali-metal assistance. These WS_2_ grains exhibited the typical triangular shapes commonly observed in solid-source CVD and MOCVD-grown WS_2_. This shape is related to the appearance of zig-zag edges in the WS_2_, which is more favourable to CVD growth than other structures, such as arm-chair edges^43^. The obtained grains were as large as ~10 μm, which is the largest grain size observed in WS_2_ films grown using H_2_S and WF_6_. The bright triangles inside the WS_2_ crystals reveal the bilayer region.

The Raman spectrum obtained from the outer region of the WS_2_ (the lower spectrum in Fig. 1c) shows two pronounced peaks, centred at 356.7 cm^−1^ (Raman active mode E′) and 418.2 cm^−1^ (A′_1_)^44–46^. A shoulder at the lower-frequency side of the E′ mode, centred at 351.5 cm^−1^, originated from the 2LA (M) mode of WS_2_^47^. The frequency difference between the E′ and A′_1_ modes was 61.5 cm^−1^, consistent with that for monolayer WS_2_^43,47^. The inner region of the WS_2_ in Fig. 1b also shows Raman peaks from the 2LA (M), ${{\rm{E}}}_{2{\rm{g}}}^{1}$, and ${{\rm{A}}}_{1{\rm{g}}}$ modes^45^ centred at 352.5, 356.5 and 419.5 cm^−1^, respectively (upper spectrum in Fig. 1c); the peak separation between the ${{\rm{E}}}_{2{\rm{g}}}^{1}$ and ${{\rm{A}}}_{1{\rm{g}}}$ modes^45^ (62.9 cm^−1^) corresponds to that of bilayer WS_2_^43,44^.

To further characterise the CVD-grown WS_2_, we measured its photoluminescence (PL) characteristics at room temperature (Fig. 1d), which revealed a single PL emissions peak at 1.969 eV. This included the emission of an A-exciton and trion from the monolayer region; the full width at half-maximum (FWHM) of the peak was 48 meV. This peak position and FWHM were similar to those of WS_2_ obtained by mechanical exfoliation or CVD growth at higher temperatures (e.g. 800 °C) using solid precursors^43,48^. Here the PL intensity from the inner region was much weaker than that from the outer region, which is consistent with a direct–indirect transition from monolayer to bilayer WS_2_^44^. The atomic force microscope (AFM) imagery, and the corresponding height profile shown in Fig. S1, confirm their monolayer and bilayer thicknesses. The obtained optical properties suggest that the crystal quality of our WS_2_ sample was similar to that of WS_2_ synthesised from solid precursors at temperatures (e.g. 800 °C) higher than those used in our CVD method (640 °C). We speculate that the successful growth of micrometre-scale, high-quality WS_2_ crystals from highly reactive, gaseous precursors is attributable to a well-controlled reaction path involving the Na additive.

The control WS_2_, grown without NaCl, did not form grains of visible size on the substrate (Fig. 1e). The corresponding scanning electron microscope (SEM) imagery and the Raman and PL spectra are shown in Fig. S2. The Raman and PL measurements indicate that WS_2_ was synthesised, but the grain size was smaller than 100 nm (Fig. S2a), which is less than 10% the size of the WS_2_ grains grown with NaCl. This result is consistent with previous findings^35^. Furthermore, observed FWHM values from the PL spectrum (Fig. S2c) for the NaCl-free control WS_2_ was 70 meV, which is much broader than that of the NaCl-assisted WS_2_, probably due to a greater abundance of defects. These results demonstrate that Na strongly suppresses nucleation and enhances the lateral growth of WS_2_ when highly reactive materials are used as precursors, leading to a pronounced improvement in crystallinity.

**Figure 2:**
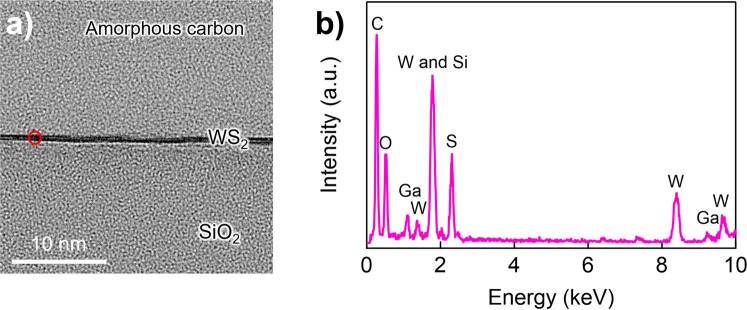
(**a**) A typical cross-sectional TEM image of WS_2_. The thickness of the WS_2_ is ~0.6 nm, which indicates that the WS_2_ is a monolayer. Amorphous carbon was deposited when the cross-sectional sample was fabricated. (**b**) The corresponding EDX spectrum measured at the WS_2_ region is marked by a red circle in (a).

Figure 2a,b show the cross-sectional transmission electron microscope (TEM) images and the corresponding energy-dispersive X-ray spectrometry (EDX) spectrum, respectively, of the WS_2_ grown with NaCl assistance. The EDX spectrum was measured at the point marked in Fig. 2a, with a spot size of ~0.1 nm. In Fig. 2b, the EDX signals from C, O, Si, S and W are labelled and clearly visible. The C, O and Si likely originated from amorphous carbon deposits on the WS_2_ surface when the TEM sample was fabricated and from the SiO_2_/Si substrate. The lack of signals associated with F, Cl and Na in the EDX spectrum indicates that the abundance of these elements was below the detectable limit for the EDX measurement (a few percent). Note that the Ga signal, which did not exist in the precursors, originated from residues associated with the focused-ion beam used to prepare the cross-sectional TEM sample.

**Figure 3:**
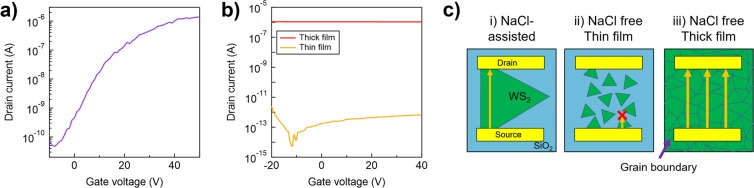
Transfer characteristics of back-gated (**a**) NaCl-assisted and (**b**) NaCl-free WS_2_ devices, as measured at a bias voltage of 10 V. (**c**) Schematics of the FET with the channel of (i) NaCl-assisted and NaCl-free (ii: thin film; iii: thick film) WS_2_. Yellow arrows show the paths of current.

The effects of NaCl were also clearly observed in the devices’ electrical properties. The WS_2_ grown with and without NaCl was fabricated into two-terminal field-effect transistors (FETs) by standard photolithography and e-beam evaporation. Figure S3 shows typical optical images of the fabricated devices. The transfer characteristics of the device fabricated with NaCl-assisted WS_2_ are shown in Fig. 3a. The device demonstrated typical n-type FET behaviour, with on/off ratio of 10^4^. This is similar to values for WS_2_ in the literature^16,49^. However, the devices fabricated with WS_2_ that was grown without NaCl showed no current modulation. Figure 3b shows the characteristics of two FET devices fabricated with NaCl-free WS_2_: one grown under the same precursor conditions as the NaCl-assisted WS_2_ and the other grown using a higher precursor concentration. The former device showed no current, while the latter device exhibited only metallic behaviour. The device with NaCl-assisted WS_2_ was prepared using a triangular-shaped WS_2_, which should not contain any grain boundaries (Fig. 3c(i)). By contrast, the grains of the NaCl-free WS_2_ were too small to merge into a continuous film (Fig. S2a) under the same growth conditions, so no connection formed between the electrodes with ~4 μm spacing and the flow of current was prevented through the channel (Fig. 3c(ii)). The NaCl-free WS_2_ grown with high precursor concentrations did form a continuous thick film (~20 nm), but the metallic behaviour of this polycrystalline WS_2_ would originate from the metallic behaviour of the grain boundaries^14^ and the impediment to carrier density modulation caused by the carrier emission from grain boundaries and the thickness of the film (Fig. 3c(iii)). A detailed discussion of this phenomenon can be found in the Supporting Information. These FET characteristics reveal another crystallinity improvement provided to WS_2_ film by the Na-assistance method.

### Grain size distribution of obtained WS_2_

**Figure 4:**
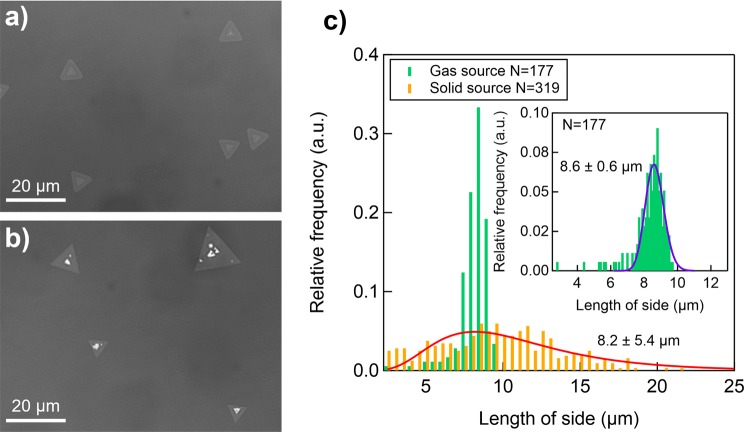
(**a**) Low-magnification optical images of (**a**) the NaCl-assisted WS_2_ and (**b**) WS_2_ grown from solid precursors at the same temperature. (**c**) The grain size distribution of the WS_2_. This distribution corresponds to an area of approximately 1 × 1.5 mm^2^ and assume that the shape of WS_2_ crystals are equilateral triangle, but results from irregular-shaped (e.g. butterfly- or star-shaped) or merged WS_2_ crystals are not included. The inset shows a magnified view of the distribution of the WS_2_ grown with gas-source CVD WS_2_.

Figure 4a shows a low-magnification optical image of the NaCl-assisted WS_2_. The domain size of this WS_2_ was substantially more uniform than that of WS_2_ prepared from solid precursors (Fig. 4b). Figure 4c shows the evaluated grain size distribution for both of these WS_2_ samples. Interestingly, the grain size distribution for the NaCl-assisted sample had a very small standard deviation (0.6 μm)—much smaller than that of the WS_2_ grown by CVD with solid precursors (5.4 μm). This small standard deviation suggests that the WS_2_ nuclei in the NaCl-assisted gas-source CVD formed simultaneously during the initial growth stage and that successive nucleation did not occur as the original nuclei grew. We speculate that this phenomenon is a consequence of using gas-phase precursors and NaCl-assistance and that the large standard deviation reported previously was due to a limited diffusion rate and non-uniformity of the solid precursor sources. For example, when sulphur vapour is raised to temperatures typically used to grow TMDs (500–1200 °C)^21,50,51^, it no longer contains atomic sulphur; it instead comprises numerous sulphur allotropes, S_n_ (2 ≤ n ≤ 8). The relatively large molecular mass and collision cross section of these allotropes lead to a low diffusion rate and easy nucleation, resulting in a large variation in grain size. By contrast, the gaseous precursors that we used have a much larger diffusion rate with a small source size (even a molecular-level source). A uniform supply and a high diffusion rate enable the sources to reach the nuclei of existing WS_2_ before aggregating, rather than forming new nuclei. Similar size uniformity has been reported for MoS_2_ grown from solid sources on hBN^52^. This uniformity could be due to the ultra-flat, dangling-bond-free surface of hBN, which promotes efficient precursor diffusion, even in the case of solid precursors with a low diffusion rate. CVD growth with gaseous precursors can suppress the unwanted nucleation of TMDs even on SiO_2_/Si substrates with dangling bonds, and it can provide conditions that enable deep exploration of the growth kinetics. Figure S5 shows Raman spectra of the WS_2_ crystals from different separated triangular grains. These results suggest uniform crystallinity across the individual grains, which were clearly observed in NaCl-assisted WS_2_. Note that this highly diffusive nature of gaseous precursors can be seen under NaCl existence, since the NaCl-free WS_2_ showed very high nucleation density (Fig. S2), indicating that the inhomogeneous nucleation occurred when NaCl was absent.

## Discussion

Now we will discuss the effect of NaCl in our gas-source CVD system. Previous studies have revealed that only cations play an important role in the alkali-metal assistance method^23^, so we have only considered the effects of Na. If Cl plays an important role in the mechanism, then CVD growth of TMDs from metal chloride (e.g. MoCl_5_) precursors should be able to obtain large-scale TMDs easily, without Na compounds. However, Song *et al*.^22^ revealed that the alkali-metal assistance method is even valid for MoS_2_ CVD growth from a MoCl_5_ precursor. Therefore, we believe that alkali metals, not chloride, drive the alkali-metal assistance method. A plausible overall chemical equation^35^ for WS_2_ synthesis from H_2_S and WF_6_ is

1 $${{\rm{WF}}}_{6}+3{{\rm{H}}}_{2}{\rm{S}}\to {{\rm{WS}}}_{2}+6{\rm{HF}}+{\rm{S}}$$

The calculated Δ*G* values for each component in Eq. (1) are shown in Table S1. In addition to the overall negative Δ*G* value for Eq. (1), the reactivity between H_2_S with WF_6_ was greater than between elemental sulphur and WO_3_, which led to a much higher reaction rate in our gas-source synthesis than in a conventional solid-source synthesis. Therefore, small WS_2_ clusters were able to form in the gas phase and function as nuclei for WS_2_. Furthermore, under a reducing atmosphere, WF_6_ can easily decompose into elemental W, as

2 $${{\rm{WF}}}_{6}+3{{\rm{H}}}_{2}{\rm{S}}\to {\rm{W}}+6{\rm{HF}}+3{\rm{S}}$$

**Figure 5:**
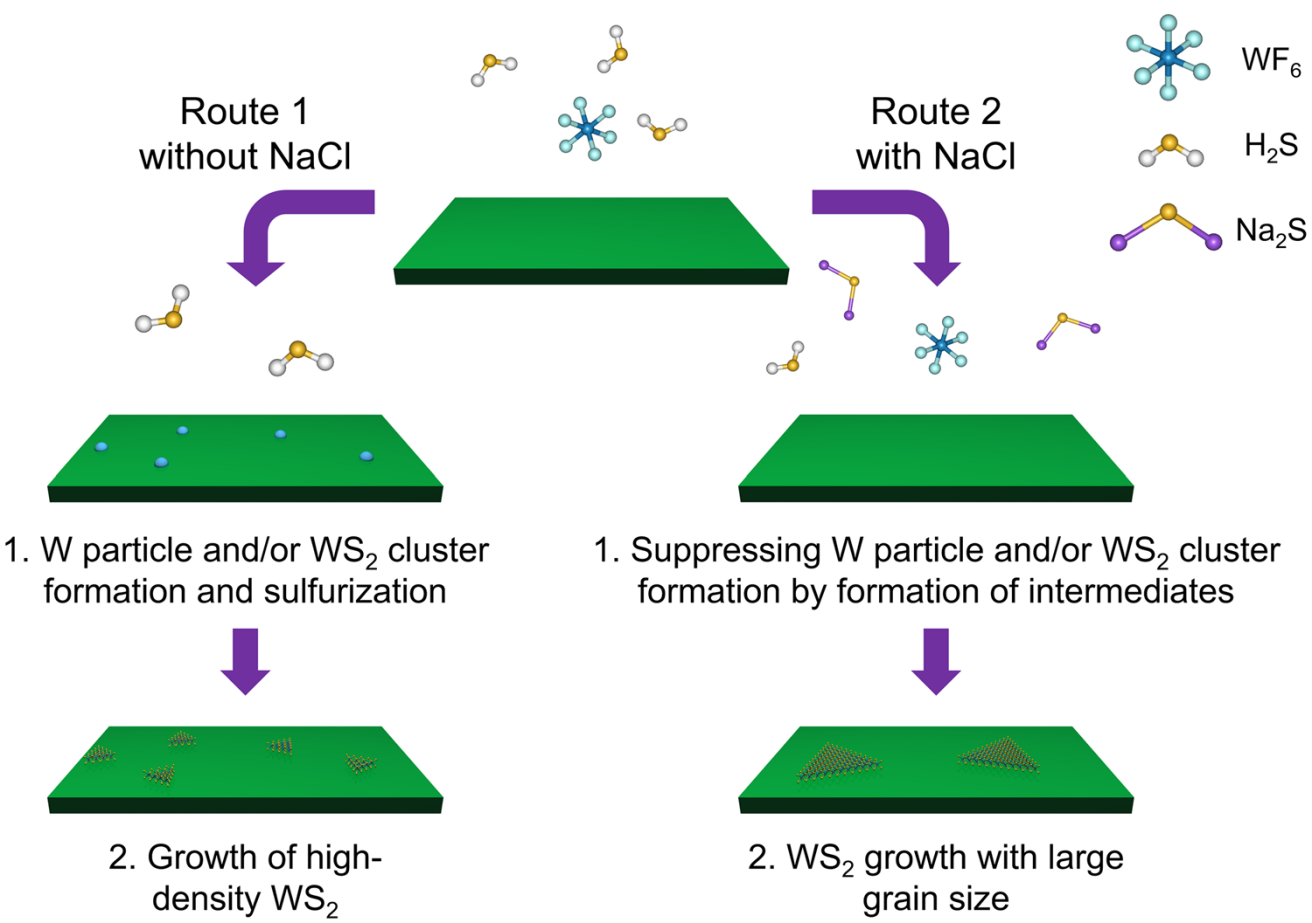
Schematic of the proposed growth mechanisms.

Because W has the second-highest melting point of all elements (~3,400 °C), the generated W should solidify and aggregate at our growth temperature, which would provide nuclei for subsequent WS_2_ growth, as represented in Fig. 5 and the equation

3 $${\rm{W}}+2{{\rm{H}}}_{2}{\rm{S}}\to {{\rm{WS}}}_{2}+2{{\rm{H}}}_{2}$$

Therefore, we propose that the poor qualities and small-domain size of the WS_2_ grown in the absence of NaCl (Fig. S2) are due to these high-density W and/or WS_2_ nuclei formed via highly active reactions. By contrast, the grain size enlargement observed when Na is added to the system suggests that it can modulate the reaction path or suppress the reaction rate through the formation of intermediate species (Fig. 5). Because our system comprises only a few elements (i.e. H, F, Na, Cl, S and W), the number of possible intermediates is limited. Several groups have recently discussed the roles of possible intermediates, such as Na_x_MoO_y_, Na_2_SiO_3_, WO_2_Cl_2_ and Na_2_S_*x*_. These intermediates are believed to enhance the diffusion and/or the wettability of the metal precursors and to decrease the energy necessary for the lateral growth of TMDs^21–27^. Na_2_S_*x*_ chains formation is the most plausible explanation in our experiment because the precursors used here do not contain oxygen. A possible reaction for this process is

4 $${{\rm{WF}}}_{6}+3{{\rm{Na}}}_{2}{{\rm{S}}}_{x}\to {{\rm{WS}}}_{2}+6{\rm{NaF}}+(3x-2){\rm{S}}$$

The Na_2_S_*x*_ chain would originate from a reaction between NaCl and H_2_S, and it may decrease the energy necessary for the growth of the TMD’s atomic layers^22^. If the system follows Eq. (4) instead of Eq. (2), it should experience suppressed formation of metallic W and/or WS_2_ clusters, resulting in grain enlargement (Fig. 5). Equation (4) has a negative Δ*G* (Table S1 shows the result for *x* = 1); therefore, this reaction is also thermodynamically favourable. Another possible mechanism of the Na_2_S_*x*_ chain at the edges of grains is a behaviour similar to Co- or Ni-assisted growth. In Ni-assisted growth of TMDs, an amorphous solid-liquid-crystalline solid growth mode has been observed^53^. During Ni-assisted growth, NiS_*x*_ was formed and dissolved W and S. Excess amounts of W and S in the NiS_*x*_ were then crystallised as WS_2_. Furthermore, previous studies also show that Na_2_S_*x*_ can function as a recrystallisation catalyst^54^. Therefore, the Na_2_S_*x*_ chain can act as a recrystallization catalyst, where the chain dissolves precursors and deposits excess W and S as crystalline WS_2_. Note that another possible case in which WO_3_ or Na act as intermediates involves the reaction of the SiO_2_ that exists in the chamber and the substrate with WF_6_:

5 $$3{{\rm{SiO}}}_{2}+2{{\rm{WF}}}_{6}\to 3{{\rm{SiF}}}_{4}+2{{\rm{WO}}}_{3}$$

Equation (5) also has a negative Δ*G* value. However, this reaction is negligible, since the reactivity between SiO_2_ and WF_6_ is poor^35^. Furthermore, the surface of the substrate was not roughened after the growth, where root-mean-square roughness of the substrate was changed from 0.197 to 0.205 nm with the growth. This result indicates that SiO_2_ did not react during the growth. Therefore, we have concluded that the SiO_2_ does not affect the WS_2_ growth even in our case. Modulating the growth reaction path would be expected to narrow the grain size distribution. As discussed before, fast reactions between WF_6_ and H_2_S cause heterogeneous nucleation of W and/or WS_2_ clusters. Therefore, the reactivity of the precursors hinders the advantages of gaseous precursors, suppressing the homogeneous nucleation through high diffusivity of the precursor. On the other hand, when NaCl is present, the modulated reaction path suppresses inhomogeneous nucleation, resulting in high precursor diffusivity. We observed the result of this process: the narrow grain size distribution.

These results shed light on one mechanism by which alkali-metal assistance functions during solid-source CVD. Prominent hypotheses are that the alkali metals increase the mass-flux by forming intermediates or that they promote the lateral growth of TMD once it has nucleated^21,23,25^. We think these two effects coexist in the solid-source CVD. Metal oxides react with alkali-metal halides to form intermediates, such as metal oxyhalides or Na-containing eutectic intermediates, resulting in an increased mass-flux rate for the metal precursors. Note that there are multiple methods to supply alkali-metal compounds: mixing a metal oxide and an alkali-metal compound^21^ or putting an alkali-metal compound on the upstream side of a metal oxide^20^. We think that NaCl should behave similarly in both cases. However, these two cases may experience different patterns of intermediate formation. When the NaCl and the metal precursor are separated, the metal precursor is available to react with NaCl vapour; when they are mixed, they react directly along their contact surfaces. Regardless of the mechanism, volatile intermediates were formed during growth and supplied into the substrate, so NaCl should function similarly. As discussed before, Na must also promote the lateral growth of TMDs, since alkali-metal assistance is even valid for CVD growth with volatile transition-metal halide precursors (e.g. MoCl_5_^22^ and WF_6_).

**Figure 6:**
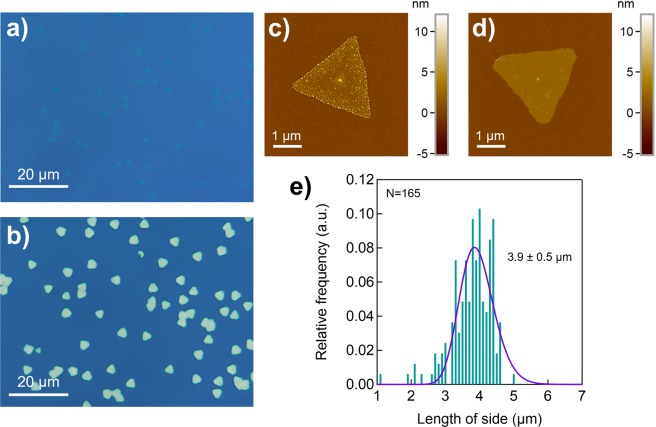
Optical images of typical NaCl-assisted WS_2_ prepared with a growth time of (**a**) 15 min or (**b**) 60 min. Neither the contrast nor the colour balance of these images was modified. (**c**) A typical AFM image of WS_2_. (**d**) A typical AFM image of WS_2_ after the specimen was immersed in deionized water for 10 min. (**e**) Grain size distribution of bilayer WS_2_. This distribution was constructed from the image shown in Fig. 4(c).

Another interesting phenomenon in our CVD growth is the switch from a 2D to a 3D growth mode during synthesis. Figure 6a,b show typical optical images of CVD-grown WS_2_ with after 15 and 60 min of growth time, respectively. As is evident in the images, WS_2_ does not form a continuous film with continuous 2D growth, but rather forms thick, bulky, isolated grains with a uniform grain size. An AFM measurement (Fig. S6) revealed that the thickness of the bulk crystal is greater than 10 nm, which corresponds to more than 10 layers. In general, Wulff’s construction for the equilibrium of crystals suggests that the large surface energy anisotropy between the *c*-axis and the other axes in TMDs, due to interlayer van der Waals forces and intralayer covalent bonds, strongly promotes the 2D growth of TMDs^55^. As a result, for typical CVD growth of TMDs, longer growth times are associated with laterally extensive TMD grains and the eventual formation of a uniform thin film^29^. However, in our CVD method, this growth mechanism is not valid; the growth mode changes from 2D to 3D without forming another new nucleus outside the initial grains. We speculate that this phenomenon is attributable to the deactivation of the edge of the WS_2_ grains and to the early layer-by-layer growth that results. As shown in the AFM image (Fig. S1 and Fig. 6c), the WS_2_ crystal was surrounded by a large number of particles on its edges and surface. Because these particles were soluble in water (Fig. 6c,d) and amount of Na and F was decreased after washing (Fig. S7), they may be water-soluble Na and F compounds, such as NaF, Na_2_WO_4_ or Na_2_CO_3_. Therefore, we have concluded that these particles are Na- and F- containing byproducts (like Na_2_WF_*x*_) or unreacted precursors of CVD growth. Because the samples were exposed to air after growth, these particles should have been oxidised, or reacted with carbon dioxide or water present in the air. We propose that these excess W and/or Na compounds, which originate from unreacted precursors, inhibit the lateral growth of WS_2_ at some point during the process. The formation of these particles would have originated from a decreased Na supply during CVD. As shown in Fig. S8, WS_2_ had largely coated the NaCl powder after CVD growth with a growth time of 60 minutes. The accumulation of this coating decreased NaCl supply gradually during the growth process. As a result, at some point, the edges of the crystal could not maintain enough Na to convert all supplied precursors into WS_2_. At that, byproducts or unreacted precursors were able to accumulate at the edges. These accumulated byproducts or unwanted precursors inhibited 2D growth and triggered the change from 2D to 3D growth. Interestingly, the grain size distribution in the WS_2_’s bilayer region also shows a small standard deviation (0.5 μm; Fig. 6e), indicating that the lateral growth of the monolayer region stopped at the same time throughout the chamber, as was observed with nucleation, and switched to second-layer growth. Subsequently, layer-by-layer growth from nuclei to the deactivated edge led to the formation of bulky WS_2_ crystals that maintained the same grain size distribution as that established by the first layer (Fig. S9). Although this growth mode is similar to the Volmer–Weber or Stranski–Krastanov growth mode in 3D crystals, these established modes cannot be readily applied to our results because only van der Waals interactions exist between WS_2_ and the substrate, and these interactions are too weak to accumulate large strain capable of inducing 3D growth. Since we believe that decreasing Na supply is the trigger for growth mode changing in the gas-source CVD growth, a modified system that ensures a continuous Na supply should allow the 2D WS_2_ crystals to grow without interruption.

In summary, we successfully synthesised ~10-μm atomic-layered WS_2_ crystals from gaseous precursors with the assistance of NaCl. The NaCl assistance method was found to be valid even with the highly reactive oxygen-free precursors H_2_S and WF_6_. The grains of the NaCl-assisted WS_2_ were more than 10 times larger than those grown in the absence of NaCl. The presence of intermediate Na compounds suppressed the formation of W and/or WS_2_ nuclei, which probably explains the substantial grain size improvement. Raman, PL and FET measurements showed that the quality of the obtained WS_2_ was similar to that of WS_2_ grown from a conventional metal oxide precursor at much higher temperatures. Our results also demonstrated that the WS_2_ grown by NaCl-assisted gas-source CVD showed a uniform grain size, suggesting that inhomogeneous nucleation was suppressed by the effective diffusion of the precursors allowed by alkali-metal assistance. We expect our results to provide a basis for WS_2_ growth processes to be able to control grain size and thickness and to promote a deeper understanding of the associated growth kinetics.

## Methods

### CVD growth

A SiO_2_ (300 nm)/Si substrate was used for CVD growth. Prior to growth, the substrate was oxidised in an oxygen atmosphere at 1000 °C for 60–180 min to prevent unwanted reactions between the precursors and Si exposed at the edges of the substrate. To remove any amorphous carbon layers, substrates were then dipped into an HF solution with a concentration of ~2% for 30 s. The substrates were then washed with deionized water for 30 s and dried with N_2_. Ceramic boats containing 100–400 mg NaCl powder (High Purity Chemicals) was loaded into a quartz tube with a diameter of 65 mm. The substrates were also placed in the tube, downstream of the boat. After the chamber was evacuated for 30–60 min, Ar was introduced as a carrier gas. The flow rate of the Ar was 100 sccm with a pressure of 1 kPa. The electric furnace surrounding the sample chamber was then heated to 640 °C, at which point H_2_S and WF_6_ were introduced into the quartz tube at flow rates of 0.33–0.40 and 0.025–0.030 sccm, respectively. Typical growth time was 15 to 60 min. We used the same CVD setup for growing NaCl-free WS_2_, however, in this case, NaCl powder was not introduced. The NaCl-free WS_2_ film was obtained at a growth temperature of 640–650 °C and pressure of 100–1000 Pa. The flow rates of Ar, H_2_S, and WF_6_ is 100–105, 0.33–5.00, and 0.025–0.050 sccm, respectively. The growth time was 20 min.

### Characterisation of WS_2_ samples

Optical images were taken with a standard optical microscope (Nikon Eclipse) with a 100× objective lens. Raman and PL measurements were performed using a confocal Raman microscope (Renishaw InVia) with a 488-nm continuous-wave laser source (COHERENT Sapphire LP). The Raman and PL peaks were modelled by Lorentzian and Voigt functions, respectively. Grain size distributions were measured with optical imagery with 50× objective lens using ImageJ and Igor Pro software for measuring grain areas and calculating their distribution, respectively. Peaks were modelled with log-normal distribution functions. AFM measurements were collected with a standard AFM (Park Systems NX10). A Schottky field-emission SEM; Hitachi SU5000 operating at an acceleration voltage of 20 kV was used to obtain SEM images. To verify the chemical state of the WS_2_ grown with NaCl assistance, we carried out cross-sectional TEM observations and EDX measurements with a high-resolution TEM (Hitachi H-9500 or JEOL ARM200F). X-ray photoelectron spectroscopy was carried out with Al Kα line excitation (Shimadzu KRATOS Nova).

### Device fabrication and characterisation

Standard lift-off processes were used to fabricate the back-gate FETs via photolithography and metal e-beam evaporation. First, the samples were spin-coated with a photoresist and the pattern was defined by a mask-less aligner. We then deposited contact materials of Ni and Au to thicknesses of 5 and 50 nm, respectively, via the e-beam evaporator. Lift-off was carried out in acetone. Electrical characterisation of the fabricated devices was conducted under atmospheric conditions with a semiconductor parameter analyser (Keysight B1500).

## Data Availability

The datasets used this this study are available from the corresponding authors on reasonable request.

## Supporting Information

Supporting Information
