# Supplementary material for: Gas-Source CVD Growth of Atomic Layered WS2 from WF6 and H2S Precursors with High Grain Size Uniformity
Source: Sci Rep. 2019 Nov 27;9:17678. doi: 10.1038/s41598-019-54049-6 (PMC6881408; doi:10.1038/s41598-019-54049-6)
Supplement: Supplementary file 1 — Supporting Information [file 41598_2019_54049_MOESM1_ESM.docx]

Supplementary information for

Gas-source CVD Growth of Atomic Layered WS_2_ from WF_6_ and H_2_S Precursors with High Grain Size Uniformity

Mitsuhiro Okada*^a^, Naoya Okada^b^, Wen-Hsin Chang^b^, Takahiko Endo^c^, Atsushi Ando^b^, Tetsuo Shimizu^a^, Toshitaka Kubo^a^, Yasumitsu Miyata^c^, and Toshifumi Irisawa*^b^

*^a^Nanomaterials Research Institute, National Institute of Advanced Industrial Science and Technology (AIST), 1-1-1, Higashi, Tsukuba, Ibaraki 305-8565, Japan*

*^b^Nanoelectronics Research Institute, National Institute of Advanced Industrial Science and Technology (AIST), 1-1-1, Umezono, Tsukuba, Ibaraki 305-8568, Japan*

*^c^Department of Physics, Tokyo Metropolitan University, 1-1, Minami-Osawa, Hachioji, Tokyo 192-0397, Japan*

E-mail: mi.okada@aist.go.jp, toshifumi1.irisawa@aist.go.jp


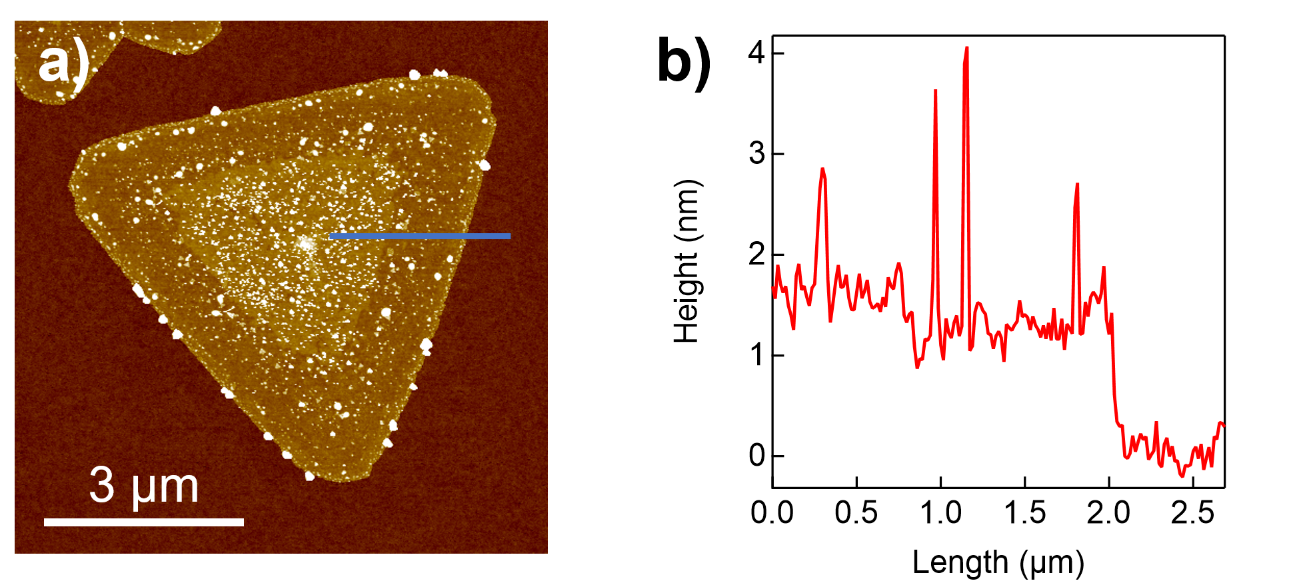


**Figure S1.** (a) an AFM topography and (b) corresponding height profile along the blue line of NaCl-assisted WS_2_. The height of obtained WS_2_ is measured as ~1 and ~1.6 nm at the inner and outer region, respectively, which clearly indicates that the WS_2_ is mono- (outer region) and bilayer (inner region).


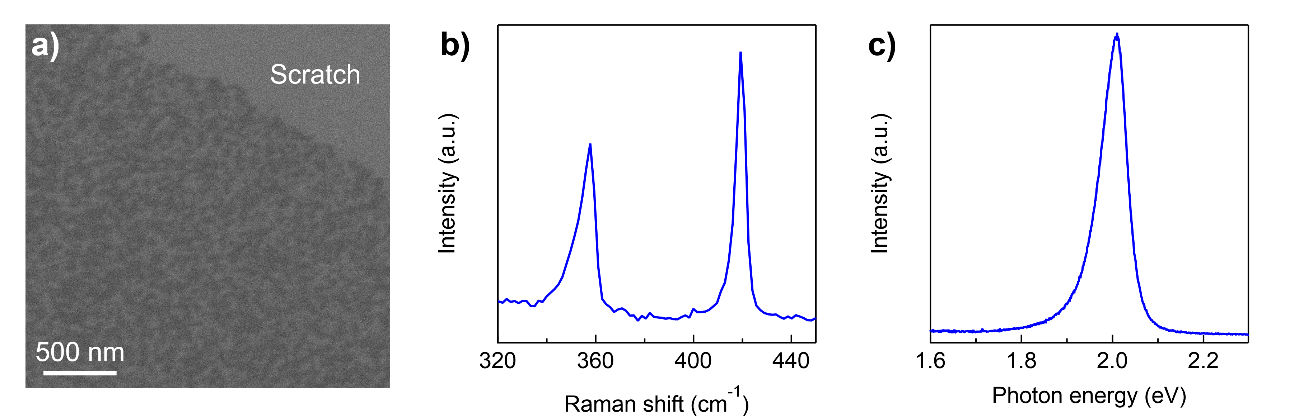


**Figure S2.** (a) a typical SEM image, (b) Raman and (c) PL spectrum of WS_2_ without NaCl-assistance. Peak separation between E´ and A´_1_ mode is measured as 61.7 cm^-1^, which corresponds to that of the monolayer.


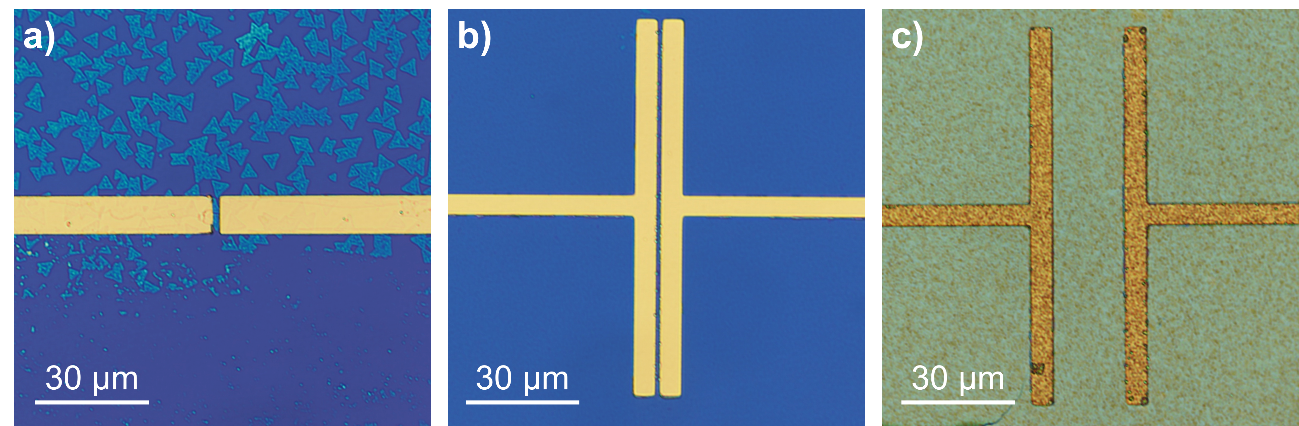


**Figure S3.** Optical images of fabricated FET using (a) a NaCl-assisted WS_2_, (b) a thin NaCl-free WS_2_ film, and (c) a thick NaCl-free WS_2_ film as a channel.

**Metallic behaviour of NaCl-free, thick WS_2_ film.**

We measured FET characteristic of other 16 devices. The results are shown in fig. S4 a): all of the devices showed metallic behaviour. The metallic behaviour on thick, NaCl-free WS_2_ film would originate from effects from grain boundaries inside. The film was made of uniform, polycrystalline WS_2_(Fig. S4 b) and c)) with a thickness of ~20 nm (Fig. S4 d) and e)), respectively. A grain size of WS_2_ seems to be less than 100 nm (Fig. S2 a)). These results indicate that there are a lot of grain boundaries between the electrode. The grain boundaries show metallic nature and emits carriers into the crystal. The former makes the FET behaviour as a metallic directly, and the latter makes the behaviour as normally-on. And the film was deposited on a SiO_2_ with a thickness of 300 nm. This SiO_2_ thickness and the thick ness of the WS_2_ film (~ 20 nm) makes current modulation by controlling Gate voltage difficult. This is because thick dielectric makes carrier modulation difficult and thick semiconductor with high carrier density keep high-conductive path even the gate voltage was applied.


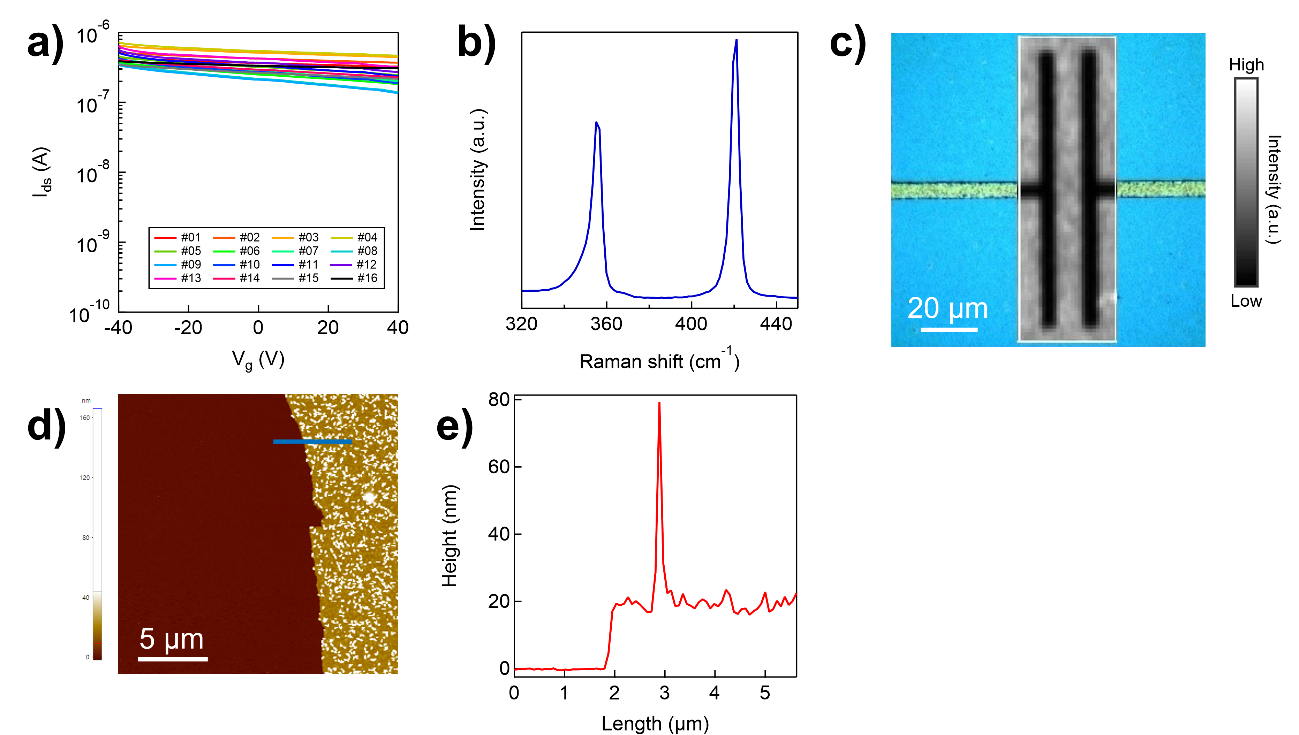


**Figure S4**. (a) FET characteristics of a polycrystalline WS_2_ film. (b) a Raman spectrum of the film. (c) a mapping image on WS_2_ A_1g_ mode intensity of a FET channel. (d) an AFM image of the film. (e) Height profile of the film along the blue line shown in (d).


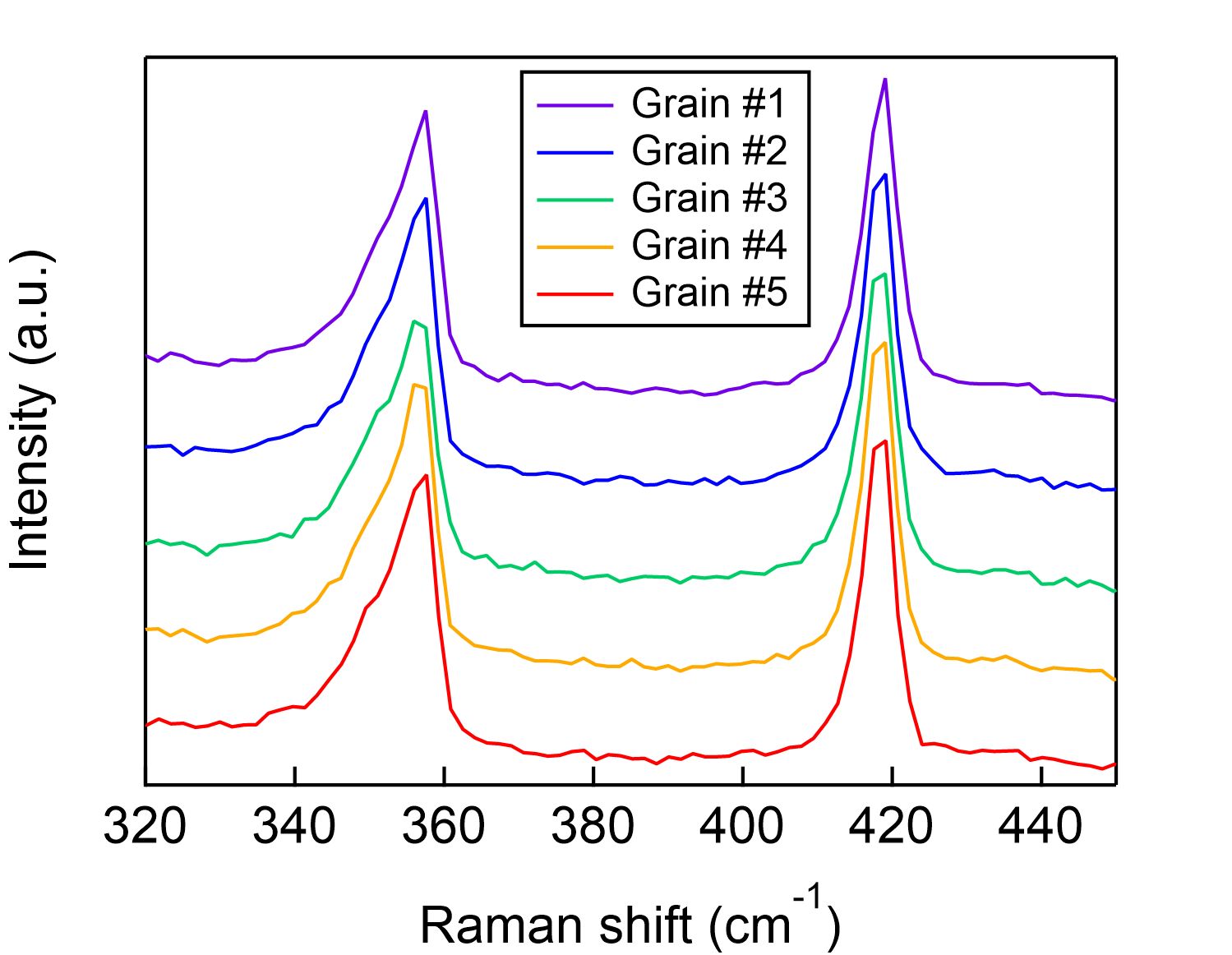


**Figure S5.** Grain-dependent Raman spectra of NaCl-assisted WS_2_.

**
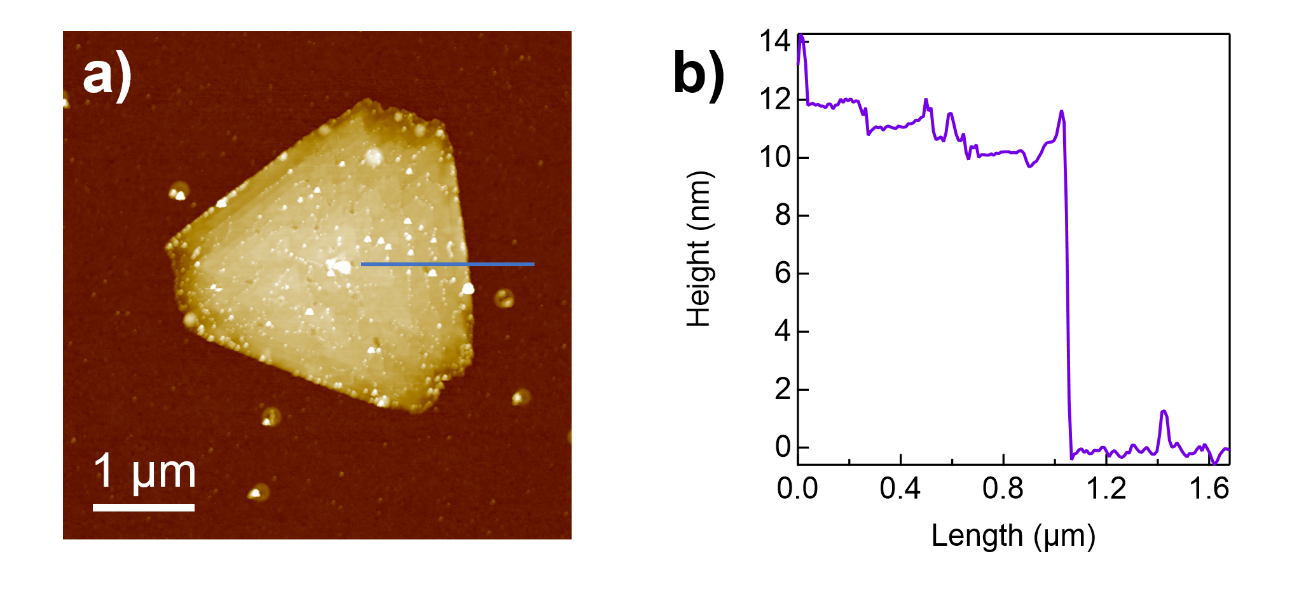
Figure S6.** (a) a typical AFM image and (b) the corresponding height profile along the blue line of an NaCl-assisted WS_2_ with a growth time of 60 minutes.


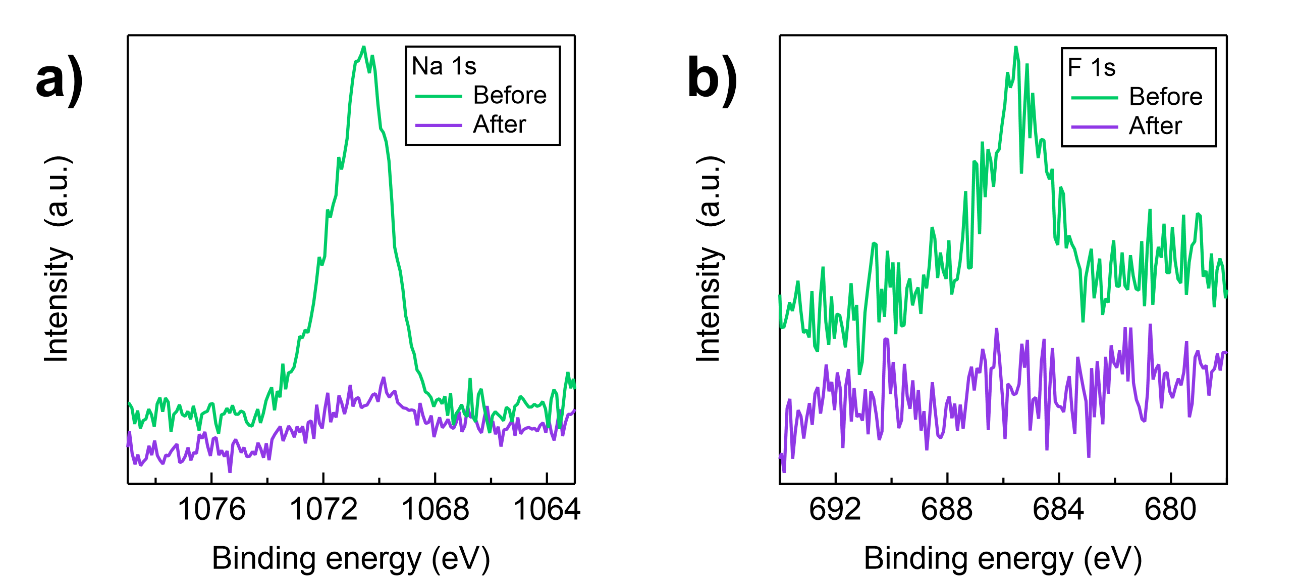


**Figure S7.** Typical X-ray photoelectron spectroscopy spectra of WS_2_ samples before and after water washing. (a) a result of a signal from Na 1s. (b) a result of signal of F 1s.


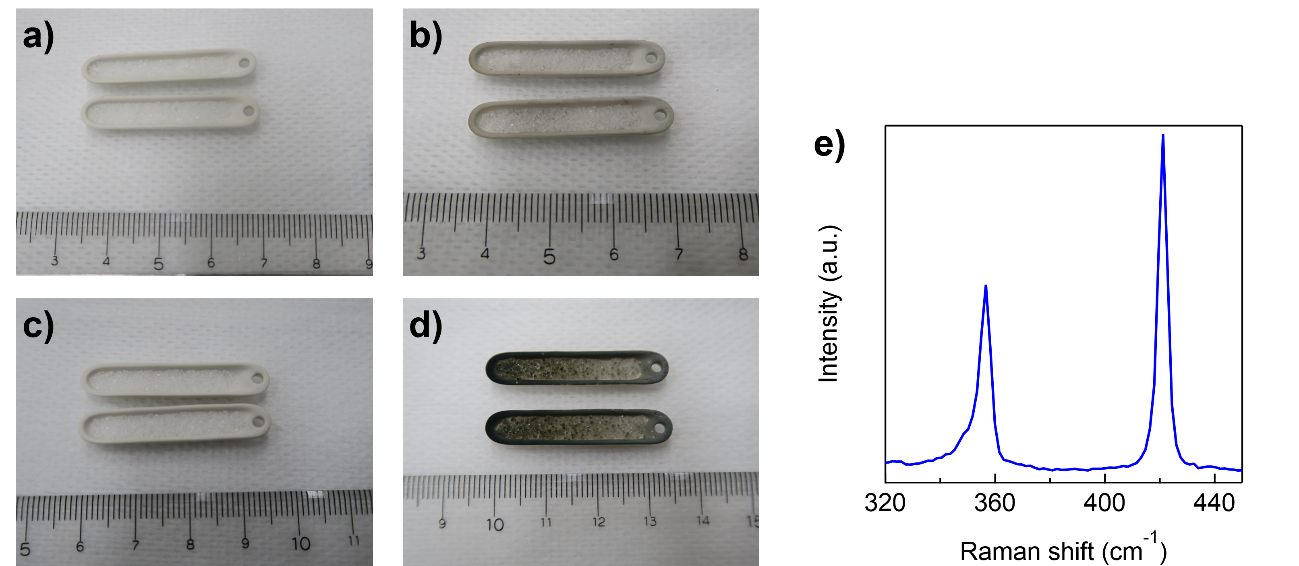


**Figure S8.** Optical images of boats used to contain NaCl. (a) before and (b) after 15 minutes growth. (c) before and (d) after 60 minutes growth. (e): A Raman spectrum of a NaCl powder obtained from (d).


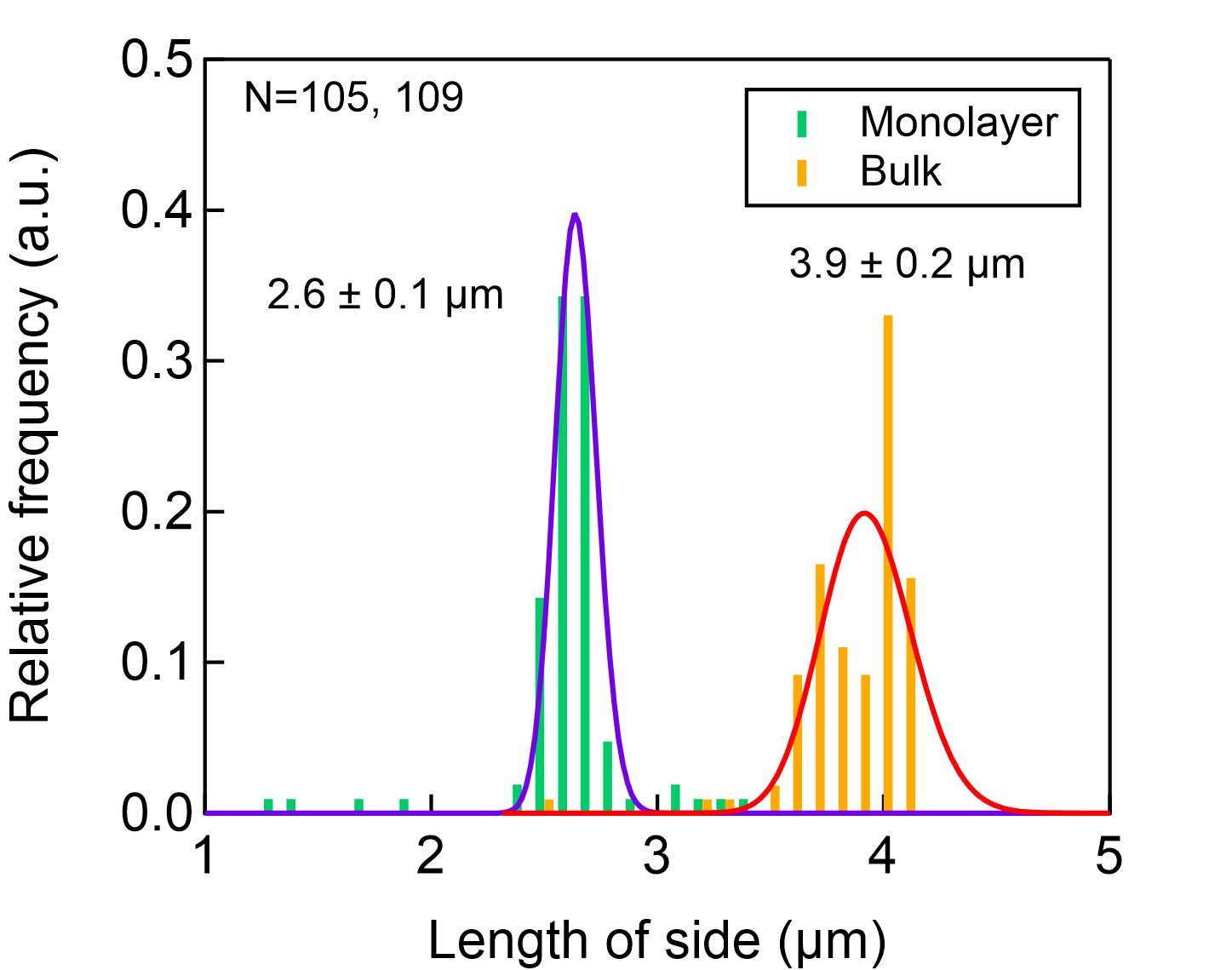


**Figure S9.** A grain size distribution of WS_2_ made from the original image of Figure 6a and 6b. Results from irregular-shaped or merged WS_2_ are not included. The length of side was calculated with an estimation of all of the WS_2_ crystals show triangular shape.

**Table S2.** Calculated Gibbs energy change in the chemical equation.

| Equation | Δ*G* (kJ/mol) | |
| --- | --- | --- |
|  | 900 K | 1000 K |
| 1 | -295.4 | -322.9 |
| 2 | -79.59 | -125.4 |
| 3 | -123.9 | -115.3 |
| 4 | -692.7 | -678.1 |
| 5 | -268.9 | -277.1 |

We used HSC Chemistry 9 for the calculation. Calculations were done with assuming that the coefficient of W compound at left side of the equation is 1.
